# X MARCKS the spot: myristoylated alanine-rich C kinase substrate in neuronal function and disease

**DOI:** 10.3389/fncel.2015.00407

**Published:** 2015-10-13

**Authors:** Jon J. Brudvig, Jill M. Weimer

**Affiliations:** ^1^Children’s Health Research Center, Sanford ResearchSioux Falls, SD, USA; ^2^Basic Biomedical Sciences, University of South DakotaVermillion, SD, USA; ^3^Department of Pediatrics, Sanford School of Medicine, University of South DakotaVermillion, SD, USA

**Keywords:** myristoylated alanine-rich C kinase substrate, actin cytoskeleton

## Abstract

Intracellular protein-protein interactions are dynamic events requiring tightly regulated spatial and temporal checkpoints. But how are these spatial and temporal cues integrated to produce highly specific molecular response patterns? A helpful analogy to this process is that of a cellular map, one based on the fleeting localization and activity of various coordinating proteins that direct a wide array of interactions between key molecules. One such protein, myristoylated alanine-rich C-kinase substrate (MARCKS) has recently emerged as an important component of this cellular map, governing a wide variety of protein interactions in every cell type within the brain. In addition to its well-documented interactions with the actin cytoskeleton, MARCKS has been found to interact with a number of other proteins involved in processes ranging from intracellular signaling to process outgrowth. Here, we will explore these diverse interactions and their role in an array of brain-specific functions that have important implications for many neurological conditions.

In the years since myristoylated alanine-rich C-kinase substrate (MARCKS) was first identified as a primary target of protein kinase C (PKC; Wu et al., 1982), it has emerged as an essential regulator of the dynamic actin cytoskeleton, membrane phosphoinositides, and many highly localized molecular interactions, with diverse roles in a variety of cell types, tissues, and organs. These roles have been heavily investigated in the brain, where the modulation of these pathways is critical for fundamental processes such as neurite outgrowth, endo and exocytosis, and synaptic plasticity. These varied functions have revealed MARCKS as an integral player in a host of physiological processes and novel etiologies, ranging from development of the cerebral cortex (Stumpo et al., 1995; Weimer et al., 2009) to aging-associated cognitive decline (Trovò et al., 2013).

MARCKS is a 32 kDa protein with two functional domains that mediate interactions with the plasma membrane (Figure 1). The N-terminus undergoes co-translational myristoylation, the covalent addition of a hydrophobic myristoyl group. Interestingly, this modification has been determined to be reversible, a phenomenon that is unique to MARCKS (Manenti et al., 1994). The effector domain (ED) is strongly basic and contains multiple serine residues that can be phosphorylated by PKC. In the unphosphorylated state, the ED has a net positive charge and is attracted to negatively charged phospholipids in the inner leaflet of the plasma membrane. This allows the N-terminal myristoyl group to reversibly insert into the plasma membrane, facilitating a relatively stable membrane interaction. When the ED is phosphorylated, however, the negatively charged phosphates reduce the affinity of the ED for the membrane, and MARCKS translocates to the cytosol (Kim et al., 1994a; McLaughlin and Aderem, 1995). Calmodulin can also associate with the ED upon activation by increased intracellular Ca^2+^ levels, similarly resulting in translocation to the cytosol (Kim et al., 1994b). This mechanism, wherein the dual interactions of the ED and the myristoyl group are required for membrane localization, has been called an “electrostatic switch” (McLaughlin and Aderem, 1995; Figure 1B). While the phosphorylation or calmodulin-mediated regulation of MARCKS localization is generally accepted in the literature, it has not been definitively demonstrated that individual MARCKS molecules repetitively cycle between cytosolic and membrane localization in response to phosphorylation and dephosphorylation. Single molecule tracking studies may soon conclusively resolve this mechanism. Furthermore, some evidence suggests that phosphorylated MARCKS may translocate not only to the cytosol, but also the nucleus, where its role is unclear (Topham et al., 1998; Michaut et al., 2005).

**Figure 1:**
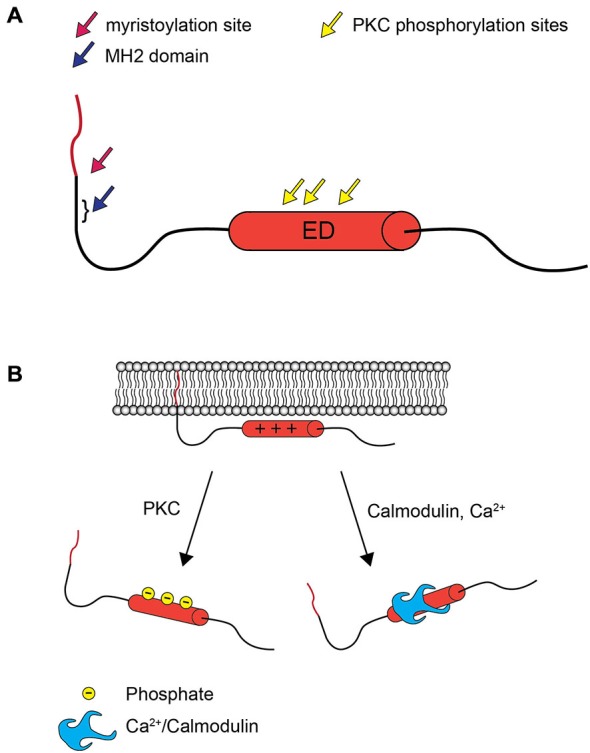
**MARCKS structure and the electrostatic switch mechanism. (A)** MARCKS contains three highly conserved domains: an N-terminal myristoylation domain, the nearby MH2 domain of unknown function, and the ED, which contains three serines that are phosphorylated by PKC. **(B)** When the ED of MARCKS is unphosphorylated, positively charged amino acid residues interact with negatively charged phospholipids in the inner leaflet of the plasma membrane, and the N-terminal myristate inserts into the plasma membrane. Phosphorylation by PKC or association with Ca^2+^/calmodulin abolishes the affinity between the ED and the plasma membrane, and MARCKS translocates to the cytosol.

PKC phosphorylation domains closely resembling the MARCKS ED, often termed MARCKS-like domains, are also found in a number of other proteins, some of which share overlapping functions with MARCKS. Diacylglycerol kinase zeta (DGKζ) is an important regulator of intracellular lipid signaling, which MARCKS is also believed to influence. DGKζ contains a MARCKS-like domain which facilitates its nuclear localization following PKC phosphorylation (Topham et al., 1998). Members of the adducin family of proteins also contain MARCKS-like domains (Joshi et al., 1991; Dong et al., 1995), are known to regulate actin and spectrin components of the cytoskeleton, and are regulated by PKC. Src-Suppressed C Kinase Substrate (SseCKS), a cell-cycle regulated scaffolding protein that interacts with membrane phospholipids and actin, contains three MARCKS-like domains which are phosphorylated by PKC (Ko et al., 2014). Another protein, aptly named MARCKS-like protein 1, maintains particularly strong homology with MARCKS, and appears to share some of its functions in CNS development (Stumpo et al., 1998). This high homology is due not only to an ED very similar to that of MARCKS, but also an N-terminal consensus myristoylation sequence (Umekage and Kato, 1991). As with the other proteins containing MARCKS-like domains, however, MARCKS-like protein 1 has differences both in terms of expression pattern and amino acid composition within and outside of the ED, resulting in distinct functional roles (Umekage and Kato, 1991; Stumpo et al., 1998).

PKC phosphorylates the MARCKS ED at three or four serines, depending on the species. In mice, where these interactions have been heavily studied, PKC phosphorylates ED serines 152, 156, and 163 (Herget et al., 1995). In addition to phosphorylation by PKC, the ED of human MARCKS is also phosphorylated by Rho-kinase at serine 159 (Ikenoya et al., 2002). MARCKS also contains unique domains outside of the ED, which are phosphorylated by proline-directed kinases at serine and threonine residues. The kinases responsible for non-ED phosphorylation include mitogen-activated protein kinase 1 (MAPK1; Schonwasser et al., 1996), cyclin-dependent kinase 1 (cdk1) and cyclin-dependent kinase 5 (cdk5; Yamamoto et al., 1995). While phosphorylation of the ED by PKC inhibits calmodulin binding, phosphorylation by cdk1 or cdk5 actually encourages it, suggesting a complex regulatory network (Yamamoto et al., 1995). Recently, the S25 residue within the relatively uncharacterized, but highly conserved, MARCKS MH2 domain was demonstrated to be phosphorylated by proline-directed kinases; primarily cdk5. This domain is only phosphorylated in neurons, does not affect association with membranes, and is yet to be functionally characterized (Tinoco et al., 2014). Interestingly, MARCKS has been demonstrated to cluster in groups with comparable phosphorylation states at the mutually exclusive S25 and ED residues (Toledo et al., 2013), perhaps due to the localization patterns induced by these modifications. The functional significance of phosphorylation outside of the ED requires further investigation; presently the vast majority of what is known regarding the regulation of MARCKS is in relation to phosphorylation by PKC.

Likewise, relatively little is known about the dephosphorylation of MARCKS, both within and outside of the ED. MARCKS is dephosphorylated, at least in part, by protein phosphatase 2A (PP2A; Tanabe et al., 2012) at both PKC and cdk1/5 residues (Yamamoto et al., 1995). Additionally, some groups have shown that calcineurin can dephosphorylate MARCKS PKC sites (Seki et al., 1995), although others have concluded that it has no such activity (Yamamoto et al., 1995). Since dephosphorylation has significant implications for the function of MARCKS, through the modulation of localization and affinity for various partners, more work is necessary in this area in order to have a balanced understanding of MARCKS regulation.

The phosphorylation-mediated regulation of membrane vs. cytosolic MARCKS localization has proven to be functionally critical. When MARCKS is localized to the membrane, the ED facilitates crosslinking of filamentous actin through two separate actin binding sites (Yarmola et al., 2001). This activity is lost upon phosphorylation of the ED (Hartwig et al., 1992), perhaps due to conformational changes induced therein (Bubb et al., 1999; Figure 2A). Thus, MARCKS is capable of transducing signals from intra- or extracellular sources, through PKC, to the actin cytoskeleton. Similarly, membrane-associated MARCKS has been shown to interact with phosphatidylinositol 4,5-bisphosphate (PIP_2_; Sundaram et al., 2004), potentially sequestering it and regulating its availability for phospholipase C (PLC) and phosphoinositide 3-kinase (PI3K), thereby regulating downstream signaling molecules such as phosphatidylinositol 3,4,5-trisphosphate (PIP_3_), diacylglycerol (DAG), and Akt (Figure 2B). This activity suggests that MARCKS could modulate sustained intracellular signaling cascades following translocation. Furthermore, MARCKS may restrict the localization of various signaling molecules and complexes, influencing cell polarity and regulating domain-specific functions (Weimer et al., 2009). In mature neurons, for example, MARCKS concentrates PIP_2_ in the postsynaptic compartment, ensuring its availability for participation in postsynaptic signaling cascades (Trovò et al., 2013).

**Figure 2:**
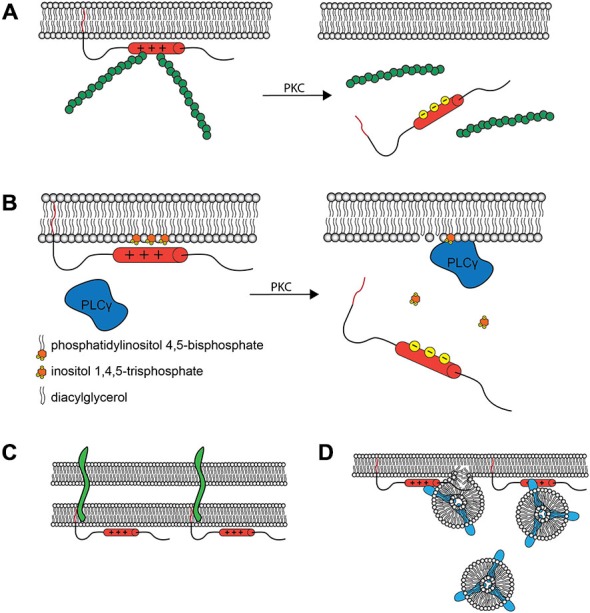
**Molecular Interactions and Cellular Roles of MARCKS. (A)** The MARCKS ED crosslinks actin filaments (green) at the plasma membrane, but this activity is reduced upon PKC phosphorylation or Ca^2+^/calmodulin binding and subsequent translocation. This is believed to facilitate morphological changes at the membrane. **(B)** MARCKS can concentrate signaling molecules such as PIP_2_ within specific membrane microdomains. Upon MARCKS translocation, these molecules are then made available for signal transduction. The influence of MARCKS on the PLCγ catalyzed production of inositol trisphosphate (IP_3_) and diacylglycerol (DAG) from PIP_2_ is one such example. In the synapse, this modulation of signal transduction is hypothesized to contribute to the role of MARCKS in learning and memory. **(C)** MARCKS interacts with PSA modified proteins, such as neural cell adhesion molecule (NCAM, green) within the plane of the plasma membrane, facilitating cell-cell interactions at the synapse and elsewhere. **(D)** MARCKS facilitates the docking and fusion of Rab10 (blue) -positive vesicles, which are hypothesized to supply the membrane necessary for process outgrowth.

As a result of its interactions with such integral structural and signaling proteins, MARCKS has been demonstrated to be involved in myriad cellular processes. In the brain, where dynamic regulation of the actin cytoskeleton plays a central role in neuronal function, MARCKS has demonstrated involvement in neural development, physiology, and neurodegeneration. This review will highlight recent advances in understanding the role of MARCKS in the brain, and will propose potential areas for future exploitation both as a window into neuronal physiology and as a therapeutic target in a variety of nervous system disorders.

## MARCKS in Brain Development

MARCKS is expressed at the highest levels in the brain during embryonic development (Stumpo et al., 1995), and ubiquitous expression persists throughout adulthood (Zhang et al., 2014). Distinct subcellular localization patterns are evident in different cell types. In neurons, MARCKS is heterogeneously distributed and enriched in axons and dendrites (Ouimet et al., 1990; Calabrese and Halpain, 2005). Different cell populations within the brain can also have widely varying expression levels; for example, MARCKS expression is robust in the hippocampal mossy fiber pathway, but not in the Schaffer collaterals (Hussain et al., 2006). During early development, MARCKS is broadly expressed in the cells surrounding the neural tube (Blackshear et al., 1996), and later, throughout the forebrain with particular enrichment at the apical membranes of ventricular zone neural progenitor cells (NPCs; Weimer et al., 2009).

Studies with *Marcks* knockout mice (*Marcks*^−/−^) have demonstrated that MARCKS is crucial for normal brain development. It has long been known that knockout is embryonically lethal, resulting in gross anatomical defects including exencephaly, lamination abnormalities, and absence of forebrain commissures (Stumpo et al., 1995). More recent investigations have begun to elucidate the cellular and molecular mechanisms underlying these defects, identifying putative functions for MARCKS in NPC proliferation, neuronal migration, and neurite outgrowth (Shiraishi et al., 2006; Weimer et al., 2009; Tanabe et al., 2012; Theis et al., 2013). Interestingly, these defects can be rescued both by mutant MARCKS lacking PKC phosphorylation sites (Scarlett and Blackshear, 2003), as well as mutant nonmyristoylatable MARCKS (Swierczynski et al., 1996).

Radial glia, which serve both as NPCs as well as a migratory scaffold for migrating neurons in the developing cortex, are displaced in *Marcks*^−/−^ embryos (Weimer et al., 2009). Radial glia typically reside adjacent to the lateral ventricles, with apical processes in the ventricular wall and basal processes extending through the cortical plate to the pial surface. The endfeet of both of these processes are concentrated with MARCKS, and it has been proposed that MARCKS serves to anchor cell polarity complexes, such as the PAR polarity complex of PAR3, PAR6, and aPKCζ, at these locations (Weimer et al., 2009). Without MARCKS, radial progenitors lose their apical attachments and become aberrantly displaced away from the ventricular zone, and do not develop appropriately oriented basal processes, rendering the migratory scaffold disorganized. Without a properly structured scaffold, clearly delineated cortical layers are lost, resulting in abundant heterotopias (Stumpo et al., 1995; Weimer et al., 2009). Retinal lamination, which is also dependent on a form of radial glia, is also defective in *Marcks*^−/−^ embryos (Stumpo et al., 1995). Rescue experiments with mutant MARCKS mice lacking either an N-terminal glycine or phosphorylatable serine residues in the ED (Stumpo et al., 1995; Swierczynski et al., 1996; Scarlett and Blackshear, 2003) have suggested that the role of MARCKS in neuronal progenitor positioning depends on myristoylation rather than phosphorylation (Weimer et al., 2009), although the specific molecular interactions underlying the functions of MARCKS in the developing cortex remain to be characterized.

It is possible that MARCKS is also influencing signaling pathways that are important for progenitor identity and differentiation. MARCKS has been shown to mediate PKC-driven modulation of ErbB2 signaling (Jin Cho et al., 2001), which is essential for maintaining radial glial identity (Ghashghaei et al., 2007). MARCKS has also recently been shown to interact with polysialic acid (PSA; Theis et al., 2013). PSA is added posttranslationally to neural cell adhesion molecule (NCAM), and has been implicated in NPC differentiation and migration (Angata et al., 2007), and axon outgrowth (Zhang et al., 1992). When intracellular MARCKS and extracellular PSA both invade the plasma membrane, they have been demonstrated to colocalize in a functional interaction in the plane of the membrane that stimulates neurite outgrowth (Theis et al., 2013; Figure 2C). Given the necessity of PSA for proper axonal commissure formation (Marx et al., 2001; Langhauser et al., 2012), these results may also hint at a molecular mechanism underlying the commissure defects observed in MARCKS knockout mice (Stumpo et al., 1995). It is also possible, however, that the defective migration patterns observed in the absence of MARCKS place newly differentiated neurons outside of their appropriate micro-niche, rendering them incapable of correctly directing axons.

Still other pathways could be responsible for the necessity of MARCKS in axon outgrowth. MARCKS has been demonstrated to interact in a phosphorylation state-dependent manner with Rab10, a small GTPase associated with plasmalemmal precursor vesicles. When the ED is in the unphosphorylated state, MARCKS mediates docking and fusion of Rab10 vesicles, which supply axons with membrane necessary for growth (Xu et al., 2014; Figure 2D). In other studies, MARCKS has also been shown to promote neurite outgrowth when dephosphorylated in response to IGF-1 stimulation (Shiraishi et al., 2006), or when phosphorylation is blocked (Gatlin et al., 2006). Taken together, these results suggest that axon outgrowth is regulated, in part, through phosphorylation/dephosphorylation of the MARCKS ED, which modulates Rab10 vesicle fusion in growth cones. This potentially contributes both to the lack of forebrain commissures and to the aberrant radial scaffold navigation observed in *Marcks*^−/−^ embryos. It remains to be seen precisely how MARCKS influences neuronal migration.

## MARCKS in the Dynamic Synapse

In the mature brain, MARCKS is enriched both within axon terminals and dendritic spines. Through interactions with actin, PIP_2_, and various signaling proteins, it has been proposed to influence synaptic signaling both pre- and postsynaptically. In presynaptic terminals, MARCKS interacts with synapsin I (Mizutani et al., 1992), colocalizes with synaptic vesicles (Ouimet et al., 1990), and is phosphorylated by PKC (Coffey et al., 1994; Obis et al., 2015). It remains to be seen, however, whether these interactions have any effect on neurotransmitter vesicle positioning or release. Thus far, electrophysiological studies have found no evidence of altered presynaptic dynamics in *Marcks*^−/−^ neurons (McNamara et al., 2005; Hussain et al., 2006). An alternative presynaptic role for MARCKS involves synapse formation and maintenance. MARCKS could aid in presynaptic growth and branching necessary for synapse formation through interactions with postsynaptic cells expressing PSA-modified proteins (Theis et al., 2013) or by modulating Rab10 vesicle localization (Xu et al., 2014): processes that have been implicated in synaptic plasticity (Muller et al., 1996; Glodowski et al., 2007). Similarly, MARCKS may be important for synaptic terminal retraction; synapses have been observed to degenerate in *Marcks*^−/−^ neurons, but the presynaptic terminals often fail to withdraw, and there is an increase in the number of terminals without a postsynaptic partner (Calabrese and Halpain, 2005). More work will be required to resolve the presynaptic effects of MARCKS.

Postsynaptic functions for MARCKS are much more firmly established. In terms of gross morphology, MARCKS overexpression causes profound changes in dendritic arborization (Calabrese and Halpain, 2005), and hyperphosphorylation of MARCKS by PKC has been correlated with reduced dendritic complexity (Garrett et al., 2012). These studies draw interesting parallels with those of other groups, who have shown that MARCKS mediates lamellipodia formation and neurite outgrowth in neuroblastoma cells (Tanabe et al., 2012), axon outgrowth in cortical neurons (Xu et al., 2014) and basal process branching in radial glia (Weimer et al., 2009). The most parsimonious explanation for these phenomena is that MARCKS is an important regulator of the fundamental actin-dependent processes required for cellular outgrowth culminating in a variety of structures. One such structure, dendritic spines, are strongly influenced by MARCKS; Knockdown or expression of a mutant form of MARCKS that mimics constitutive phosphorylation results in decreased spine number, size, and width, and over expression results in spine elongation and fusion (Calabrese and Halpain, 2005). These changes appear to be largely influenced by MARCKS-mediated translocation of f-actin. When phosphorylated, MARCKS translocates from the inner membrane surface, reducing levels of f-actin within spines, causing some to retract or fuse with neighboring spines, and others to exhibit immature filopodia-like morphology (Calabrese and Halpain, 2005). Since synapse stability and strength are largely influenced by dendritic spine morphology, with wide, bulbous spines promoting persistent synapses, these morphological changes, as well as the reduction in spine numbers, suggest that altered levels of postsynaptic membrane-associated MARCKS may cause synapse destabilization. These changes are of functional consequence. Reduced MARCKS levels have been shown to inhibit long-term potentiation (LTP) and spatial learning in mice, without affecting basal synaptic transmission (McNamara et al., 2005; Hussain et al., 2006). Unique patterns of MARCKS translocation have also been observed after imprinting training in chicks. In this paradigm, chicks are trained to recognize (and prefer) a unique moving object shortly after hatching, taking advantage of the critical window when chicks will “imprint” on the first moving object they see. Twenty four hours after training, membrane-associated MARCKS increases in the intermediate medial mesopallidum, where imprinting memories are stored (Solomonia et al., 2008). It is plausible that, during training, increases in postsynaptic calcium in learning-specific circuits activate calmodulin, which binds the ED of MARCKS resulting in its translocation to the cytosol, allowing for structural changes to take place at the membrane. Once these changes have taken place, increasing membrane-associated MARCKS then stabilizes the new spine configurations (Solomonia et al., 2008). This is a tempting hypothesis, and will require further investigation into MARCKS translocation patterns in memory acquisition, consolidation, and extinction, in which new associative memories supersede old ones.

Besides phosphorylation and calmodulin binding, what else could be regulating MARCKS at the synapse? MARCKS has been shown to be selectively degraded by cathepsin-B after intense stimulation of N-methyl-D-aspartate (NMDA) receptors, resulting in dendritic spine collapse (Graber et al., 2004). Following brief ischemic insults, postsynaptic MARCKS can also be ubiquitinated, resulting in a brief and reversible form of dendritic spine collapse that is believed to be protective against NMDA receptor-mediated toxicity (Meller et al., 2008). These results demonstrate the complex manner in which MARCKS is posttranslationally regulated. MARCKS can be phosphorylated within or outside of the ED, cleaved by cathepsin-B, myristoylated and demyristoylated, and ubiquitinated, all presumably modifying the ability of MARCKS to crosslink actin filaments, and regulating membrane and cytoskeleton plasticity in dynamic locations such as dendritic spines.

MARCKS has also been shown to influence synaptic plasticity through modulation of PIP2 availability. In aging mice, synaptic levels of membrane MARCKS have been shown to decline concurrently with PIP_2_ levels and PLCγ activity, and in culture, MARCKS overexpression is sufficient to restore synaptic PIP_2_ and PLCγ (Trovò et al., 2013). Intriguingly, MARCKS overexpression *in vivo* completely rescues age-related deficits in LTP and memory retention (Trovò et al., 2013). The authors of this study suggest that MARCKS must be sequestering PIP_2_ at specific sites in the membrane, such as dendritic spines, and then releasing it at appropriate times to allow for PIP_2_-related signaling cascades. Furthermore, their results suggest that an abundance of postsynaptic MARCKS appears to promote healthy synaptic PIP_2_ and PLCγ levels throughout life, maintaining some of the signaling cascades important for learning and memory. This activity complements the putative role of MARCKS in directly crosslinking actin within spines, as PIP_2_ itself is known as an important regulator of the actin cytoskeleton.

## MARCKS in Non-Neuronal Cells within the Brain

The diverse roles of actin and signaling molecules like PIP_2_ are not limited to synapses or neurons, and as such, MARCKS has many functions in other cell types within the brain. PKC signaling underlies some types of blood-retinal barrier breakdown (Titchenell et al., 2012), and MARCKS has been shown to regulate endothelial cell permeability through modification of the actin cytoskeleton (Jin et al., 2012), hinting at a possible role for MARCKS in blood-brain barrier (BBB) dynamics. Similarly, MARCKS appears to be important in ependymal cells, which form the interface between cerebrospinal fluid and the brain interstitium. A recent study demonstrated an exciting novel role for MARCKS in the aging brain; membrane associated MARCKS declines with age in ependymal cells, concomitant with decreasing function in mucin clearance and barrier functions (Muthusamy et al., 2015). In these cells, MARCKS influences the localization of protein chloride channel calcium-activated family member 3 (Clca3), which decorates mucin granules. Interestingly, healthy ependymal cells concentrate MARCKS at their apical membranes (Muthusamy et al., 2015), similar to the localization pattern observed in the radial glia that line the ventricles during development (Weimer et al., 2009). This apical clustering is lost, however, with advanced age in mice, resulting in the observed deficits. When MARCKS is conditionally-deleted in ependymal cells, ependymal barrier function is similarly hindered, leading to the aberrant activation of astrocytes and microglia in the forebrain interstitium (Muthusamy et al., 2015). These results underscore the possible involvement of MARCKS in a variety of aging-related etiologies.

MARCKS also has roles in glial cells themselves. In microglia, MARCKS mRNA and protein are upregulated in response to lipopolysaccharide (LPS; Sunohara et al., 2001) or amyloid-β (Aβ; Hasegawa et al., 2001; Murphy et al., 2003) administration, and kainic acid-induced seizures (Eun et al., 2006): all treatments that result in microglial activation. In astrocytes, MARCKS has been shown to participate in a pathway that regulates migration and morphological changes resembling reactive gliosis (Lee et al., 2012). MARCKS has also been shown to regulate the differentiation of oligodendrocytes in a process that involves both reorganization of actin networks and the polarization of trafficking cues (Baron et al., 1999). These findings demonstrate the ubiquitous importance of MARCKS in the brain, and support a need for further research examining the functions of MARCKS in endothelial and various glial cells.

## MARCKS in Neurological Disorders

Given its critical roles in a variety of brain cell types, it is not surprising that MARCKS has been the subject of intense research into the etiology and treatment of various neurological disorders. Results thus far have been encouraging, identifying MARCKS as an important player in numerous disorders involving synaptic abnormalities and neurodegeneration (Lenox and Wang, 2003; Kim et al., 2010; Yamamoto et al., 2014; Tagawa et al., 2015; Trovò et al., 2015), and investigation is underway to identify treatments that will effectively modulate MARCKS activity to restore physiological functions (Singer et al., 2004).

There are multiple ways in which MARCKS levels could be modulated in human disease, including through gene polymorphisms. It is unknown how widespread MARCKS polymorphisms are in humans. Results with animal models suggest that loss of function mutations would be embryonically lethal in the homozygous condition (Stumpo et al., 1995), although this has never been confirmed in human patients. Heterozygotes, however, would be much more likely to survive and may possess some of the deficiencies observed in *Marcks*^+/−^ mice, such as impaired LTP and spatial learning (McNamara et al., 1998; Hussain et al., 2006). The prevalence of MARCKS mutations in humans and associations with diseases including learning disorders warrants further attention.

Even in the absence of *MARCKS* mutations, altered MARCKS activity has been found to be part of the core etiology in some genetic conditions. Niemann-Pick disease type A (NPDA), a fatal lysosomal storage disorder caused by mutations in the acid sphingomyelinase gene, results in the accumulation of sphingomyelin in lysosomes and the plasma membrane which, in turn, leads to reduced synaptic levels of MARCKS and PIP_2_, and synaptic deficits. Intracerebroventricular infusion of recombinant MARCKS ED restores PIP_2_ levels and improves behavioral parameters in mouse models of NPDA (Trovò et al., 2015). Another condition, spinocerebellar ataxia type 14 (SA), is caused by mutations in PKCγ. Cell culture studies have shown that the mutant PKCγ inhibits synaptogenesis and dendrite development (Seki et al., 2009). The investigators who performed this work hypothesized that a deficit in macropinocytosis, a process in which endocytosis is used to traffic membranes, underlies these problems, and have demonstrated that MARCKS may be the relevant PKCγ substrate dysregulated in the disease state (Yamamoto et al., 2014). Going beyond SA, these findings support a general role for MARCKS in endocytosis, which is not surprising, considering the MARCKS ED has been shown to have membrane curvature sensing capability (Morton et al., 2014). Intriguingly, Other authors have suggested that MARCKS-mediated endocytosis deficits could contribute to amyloid beta aggregation in Alzheimer’s disease (AD; Su et al., 2010).

Additionally, altered MARCKS levels have been implicated in a number of mental disorders, and have been observed in schizophrenia (Pinner et al., 2014) and depression (Redei et al., 2014) patients, as well as violent suicide completers (Punzi et al., 2014). It is unclear, however, if these observations represent transcriptional, translational, or other aberrations. Surprisingly, it is possible that MARCKS has unknowingly been targeted for decades in the treatment of psychiatric disorders. One study demonstrates that electroconvulsive therapy (ECT) increases phosphorylation of MARCKS in the time period immediately following treatment, potentially facilitating the long-term synaptic changes that make ECT effective (Kim et al., 2010). MARCKS is also responsive to lithium treatment. Chronic lithium administration acts as a repressor at the MARCKS promoter, decreasing brain mRNA and protein levels by 50%, and has been proposed to be, at least partially, responsible for the efficacy of lithium treatment in bipolar disorder (Lenox and Wang, 2003). Interestingly, lithium also has some demonstrated efficacy in neurodegenerative disorders where MARCKS involvement has been proposed, namely Parkinson’s disease (PD; Kim et al., 2011; Lieu et al., 2014) and AD (Appleby and Cummings, 2013; Sofola-Adesakin et al., 2014).

A microarray study identified *MARCKS* as one of four neurotransmission-related genes differentially expressed in PD (Rakshit et al., 2014). Likewise, a recent study found a strong association between MARCKS phosphorylation and AD pathology in both humans and mice, and determined that hyperphosphorylation of MARCKS was the primary factor in the initiation of AD synapse pathology. Remarkably, the same study found that shRNA-mediated knockdown of MARCKS was able to rescue decreased spine density in cortical neurons in a mouse model of AD (Tagawa et al., 2015). This is a surprising result, considering that the hyperphosphorylation observed in AD would be expected to *reduce* the amount of MARCKS available at the membrane for actin crosslinking and PIP_2_ sequestration. The authors were working under the unconventional hypothesis that phosphorylated MARCKS actively inhibits actin crosslinking, rather than unphosphorylated MARCKS facilitating it. Within this model, MARCKS knockdown could allow polymerized actin to stabilize dendritic spines, leading to the observed outcomes. Still, these results are in direct contrast with those of other authors, including Calabrese and Halpain (2005) who found that MARCKS knockdown *reduces* spine density. These conflicting results will require resolution with more studies.

Nevertheless, the work of Tagawa *et al*. provides convincing evidence for the presence of MARCKS etiology in AD, an idea which is supported by the work of several groups. Phosphorylated MARCKS has been observed within dystrophic neurites and senile plaques in the brains of human AD patients (Kimura et al., 2000). Aβ, the main component of senile plaques and the putative toxic species underlying AD pathology, causes MARCKS phosphorylation through PKC and MAPK, as well as MARCKS upregulation, when applied exogenously in cultured neurons and microglia (Hasegawa et al., 2001; Tanimukai et al., 2002; Murphy et al., 2003). MARCKS has also been implicated in the secretion of apolipoprotein E (apoE; Karunakaran et al., 2013), a cholesterol transporting molecule with certain polymorphisms strongly associated with AD.

The associations with AD and roles in dendritic spine maintenance also suggest possible roles for MARCKS in Down syndrome (DS). DS, caused by trisomy of chromosome 21, is frequently comorbid with AD. Neocortical lamination defects and dendritic spine abnormalities have been observed in human DS patients (Kaufmann and Moser, 2000, in review), and reductions in dendritic spines are more severe when DS and AD are comorbid (Ferrer and Gullotta, 1990). These features resemble some of those seen in studies where MARCKS levels and activity are altered (Calabrese and Halpain, 2005; Weimer et al., 2009). The gene for amyloid precursor protein (APP) is located on chromosome 21, which has been proposed to explain the high prevalence of AD in DS patients. Interestingly, a *MARCKS* pseudogene, *MARCKSP1*, is located adjacent to and approximately 56 kilobases upstream from, the APP gene. When *MARCKS* was first mapped to chromosome 6, it was observed that MARCKS mRNA hybridized strongly to a location on chromosome 21 (Sakai et al., 1992); presumably this was the *MARCKS* pseudogene. Could the expression of MARCKS and APP be linked through this pseudogene? It is unknown if *MARCKSP1* is transcribed, but, if it is, it is possible that its transcription could be coregulated with that of APP due to proximity and shared cis-acting elements. Interestingly, *MARCKSP1* contains a microRNA-21 targeting site identical to one found in the 3’ untranslated region of *MARCKS*. Since microRNA-21 has been shown to regulate MARCKS (Li et al., 2009), if a *MARCKSP1* transcript were produced it could potentially act as a microRNA sponge to influence MARCKS expression. While only speculative, such a scenario could have important implications for AD and DS. Future studies should investigate genetic interactions such as these, as well as potential roles for MARCKS in DS and other cognitive disorders involving dendritic spine pathology.

Likewise, MARCKS etiology and potential as a treatment target should be explored in other neurodegenerative diseases, such as amyotrophic lateral sclerosis (ALS) and Huntington’s disease, where dendritic spine loss has been observed to preclude neurodegeneration. To date, no studies have been published examining the role of MARCKS in these disorders. MARCKS also has demonstrated involvement in a number of other conditions where it has not been adequately investigated, including neuropathic pain (Tatsumi et al., 2005), seizure disorders (Graber et al., 2004; Eun et al., 2006), gliomas (Jarboe et al., 2012), and methyl mercury poisoning (Shiraishi et al., 2014).

## Future Directions

The last three decades have seen great progress towards understanding the structure, regulation, and functions of MARCKS. Much remains unknown, however. At the fundamental level, the nature of the interactions between MARCKS and its various protein partners, such as actin and PKC, are still somewhat unclear. Much evidence supports the notion that MARCKS influences the actin cytoskeleton, but the molecular details of this interaction, such as specific binding sites and responsivity to specific phosphorylation patterns, require some clarification. Similarly, while MARCKS is a well-established substrate for PKC, this function appears to go beyond a simple kinase-substrate relationship (Yamamoto et al., 2014), with the two proteins actively influencing the localization of one another and thus, their availability for other partners. A better understanding of these processes should help reconcile some of the conflicting results that have been obtained to date, and provide insight into other cellular roles for MARCKS.

There is strong evidence that MARKS has different functions in different cell types and at different times in development (Solomonia et al., 2008; Weimer et al., 2009; Trovò et al., 2013; Muthusamy et al., 2015). These variable functions could be largely mediated through subcellular localization patterns of MARCKS, but also through its ability to dock and bind different partners. Characterizing these variable functions, as well as the specific interactions that facilitate them, remains an important goal. Studies using conditional and inducible genetic manipulations targeting MARCKS will enable great progress in dissecting these functions and pathways. Likewise, animal models such as the newly described floxed *Marcks* mouse (Muthusamy et al., 2015), will allow the effects of MARCKS knockout to be studied *in vivo* in discrete cell types through time points beyond birth, circumventing the lethality of complete knockout.

Even within specific cell populations, it appears likely that MARCKS could have a multitude of roles. While the functions of MARCKS have been most extensively studied in cell periphery, in relation to cytoskeletal and phosphoinositide dynamics, MARCKS has also frequently been observed in the perinuclear region (Techasen et al., 2010; Yamamoto et al., 2014), where its function is not well understood, and even in the nucleus itself, in the case of oocytes (Michaut et al., 2005). While nuclear localization has not been reported in other cell types, it may be expected to occur to some extent, given the presence of a nuclear localization signal within the ED (Topham et al., 1998). What could MARCKS be doing in the nucleus? Future studies should examine possible roles in nuclear phosphoinositide signaling, nuclear actin modulation, and even potential transcription factor activity.

Within all localizations, the regulation of MARCKS activity also requires further investigation. Phosphorylation by PKC has been relatively well characterized, but the influence of other kinases that phosphorylate MARCKS both within and outside of the ED should be explored. Non-kinase regulatory mechanisms such as myristoylation/demyristoylation cycles and ubiquitination have only begun to be investigated, as have mechanisms regulating transcription and mRNA stability. For example, micro-RNA 21 has been shown to target MARCKS (Li et al., 2009), and a long noncoding RNA transcribed from a region adjacent to *MARCKS* has been shown to influence methylation of *MARCKS* and is upregulated in certain conditions (Punzi et al., 2014), but interactions like this one are not well understood. Similarly, while the MARCKS promoter contains a number of transcription factor binding sites (Wang et al., 2002), the significance of these interactions has not been elucidated.

Functionally, MARCKS is proving to have important implications in a variety of conditions. In addition to various neurodegenerative and cognitive disorders, several studies have recently demonstrated that the declining MARCKS levels that occur with age may underlie some forms of aging-related pathology. Although this review focused only on brain-related functions and disorders, MARCKS is also the subject of intense investigation in other organ systems, and this work has already resulted in promising therapies for some conditions (Singer et al., 2004). In the coming years, improved knowledge of MARCKS function has great potential to generate better models of basic physiological and pathological processes, as well as novel therapies.

## Funding

This work was supported in part by NIH: R01NS082283 (JMW).

## Conflict of Interest Statement

The authors declare that the research was conducted in the absence of any commercial or financial relationships that could be construed as a potential conflict of interest.
